# Targeting Redox Homeostasis and Cell Survival Signaling with a Flavonoid-Rich Extract of Bergamot Juice in In Vitro and In Vivo Colorectal Cancer Models

**DOI:** 10.3390/pharmaceutics18010007

**Published:** 2025-12-20

**Authors:** Alessandro Maugeri, Paola De Cicco, Rebecca Amico, Martina Farina, Michele Navarra, Francesca Borrelli

**Affiliations:** 1 Department of Veterinary Sciences, University of Messina, I-98168 Messina, Italy; amaugeri@unime.it; 2 Department of Pharmacy, School of Medicine and Surgery, University of Naples Federico II, I-80131 Naples, Italy; paola.decicco@unina.it (P.D.C.); rebecca.amico@unina.it (R.A.); franborr@unina.it (F.B.); 3 Department of Chemical, Biological, Pharmaceutical and Environmental Sciences, University of Messina, I-98166 Messina, Italy; mfarina@unime.it

**Keywords:** colorectal cancer, nutraceuticals, flavonoids, bergamot, *Citrus bergamia*, HCT-116, azoxymethane, mice

## Abstract

**Background/Objectives**: Colorectal cancer (CRC) is the second most common cause of cancer death worldwide. Evidence suggests that a polyphenol-rich diet may lower the risk of CRC. The aim of this study was to demonstrate the potential antitumor effects of a flavonoid-rich extract of bergamot juice (BJe) in both in vitro and in vivo CRC models, assessing the underlying mechanisms. **Methods**: CRC cells, among which HCT-116, have been employed to assess the fine mechanism of action of BJe, whereas a mouse model of azoxymethane (AOM)-induced CRC was exploited to appreciate the anti-cancer effects of BJe. **Results**: BJe inhibited the growth of several CRC cells, especially HCT-116. In this cell line, BJe induced apoptosis and blocked the cell cycle in the G1 phase, as well as modulated the gene expression of apoptosis- and cell cycle-related factors. Moreover, BJe prompted reactive oxygen species production and impaired mitochondrial membrane potential. In the nucleus of these cancerous cells, BJe induced DNA damage as confirmed by the raised levels of 8-oxo-2′-deoxyguanosine and phosphorylation of histone H2A.X. In mice with AOM-induced CRC, BJe was able to lower the number of aberrant crypt foci. Moreover, BJe reduced the percentage of mice bearing both polyps and tumors, as well as their number. **Conclusions**: Our study supports the role of BJe against CRC, providing knowledge on the underlying mechanism of action.

## 1. Introduction

According to the latest global cancer statistics (GLOBOCAN), colorectal cancer (CRC) was the third most frequently detected neoplasm worldwide in 2022, after lung and breast cancers, representing the second leading cause of death from cancer. In particular, it has been estimated that around 2 million new cases of CRC have been diagnosed in the reference year, with more than 900,000 deaths [1]. Regardless of gender, CRC ranks third for both incidence and mortality in males and females [2]. Due to lifestyle factors, the highest occurrence rates are reported in countries with a high human development index (HDI). As a matter of fact, urbanization and westernization are contributing to an increase in CRC rates in low- to middle-HDI countries [3,4]. Among these factors, diet plays a pivotal role as evidenced by several epidemiologic investigations that have associated the consumption of certain foods (e.g., red meats, processed foods, etc.) with the risk of CRC [5]. In contrast, diets rich in fruits and vegetables are acknowledged for their beneficial role against several neoplasms, due to their high content of polyphenols [6,7]. Numerous biological mechanisms, such as cell-cycle regulation, apoptosis induction, hormone regulation, anti-inflammatory, and antioxidative effects, as well as anti-angiogenic properties, have been shown to support the effectiveness of polyphenols in preventing a wide range of diseases [8,9], among which is CRC [10,11,12].

One of the most relevant sources of polyphenols in healthy dietary regimens is citrus fruits (CFs) [13]. Indeed, the consumption of CF has been inversely correlated with several neoplasms, like those affecting the lungs [14], esophagus [15], and stomach [16]. Among CFs, the fruit of *Citrus* × *bergamia* Risso (bergamot) is mainly acknowledged for the extraction of the essential oil, which is commercially exploited in the perfume industry. Nevertheless, in the last few decades, bergamot derivatives have been investigated for their pharmacological properties, among which are anti-cancer ones. Indeed, the juice (BJ) proved to possess interesting anti-proliferative and proapoptotic effects in hepatocellular carcinoma cells [17]. Deeper investigations have ascribed to the flavonoid-rich extract of BJ (BJe) the key role in anticancer effects of bergamot, as it has been documented both in vitro and in vivo models [18,19]. BJe is a phytocomplex characterized by the presence of flavanone glycosides like neohesperidin, naringin, melitidin, and neoeriocitrin, which present hydroxy and methoxy groups in the backbone scaffold, as well as variously substituted sugar moieties [18].

Therefore, the aim of this study was to investigate the anti-cancer properties of BJe in both in vitro and in vivo models of CRC, assessing the underlying mechanisms of action.

## 2. Materials and Methods

### 2.1. Bergamot Juice Extract (BJe)

BJe was generously gifted by the Herbal and Antioxidant Derivatives Srl (Bianco, Reggio Calabria, Italy), which was obtained as described [20]. Briefly, the juice was obtained from unpeeled fruits through industrial pressing. Afterwards, it was loaded on appropriate polystyrene resin columns to absorb polyphenol compounds. The elution of the flavonoid fraction was performed by a mild basic solution, neutralized by filtration on cationic resin at an acidic pH. Finally, the resulting eluate was vacuum-dried to achieve BJe [20].

The quali-quantitative chemical composition of BJe was investigated through HPLC analysis performed using a Fast 1200 HPLC system (Agilent Technologies, Santa Clara, CA, USA) equipped with a DAD detector. A gradient of two solvents, water and acetonitrile, was employed to elute the sample, which was injected into a volume of 2 μL of ethanol. The flow rate was 3 mL min^−1^, and the ZORBAX eclipse XDB-C18 column (50 mm) was maintained at 35 °C. The diode array detector was monitored at 280 nm. Flavonoid pure standards were purchased from Sigma-Aldrich (Burlington, MA, USA) [20].

### 2.2. Cell Cultures

The human colon carcinoma HT-29 and Caco-2 cells were cultured in RPMI 1640 medium, while HCT-116 cells were cultured in low-glucose DMEM (plus 2.5% HEPES and 1% of non-essential amino acid (NEAA). Cells were originally obtained from ATCC (Rockville, MD, USA). Both media were supplemented with 10% heat-inactivated fetal bovine serum (FBS), penicillin and streptomycin (100 IU/mL and 100 µg/mL, respectively), along with L-glutamine (2 mM) and sodium pyruvate (1 mM). Cells were maintained at 37 °C in a 5% CO_2_ air-humidified atmosphere. The reagents for cell growth were obtained from EuroClone (Milan, Italy).

### 2.3. Cell Proliferation Assay

Cell proliferation was assessed by the 3-(4,5-dimethylthiazol-2-yl)-2,5-diphenyltetrazolium bromide (MTT) test, as previously described [17]. Briefly, cells were seeded in 96-well plates (1 × 10^4^ cells/well). After 24 h, cells were treated with BJe (0.25–5 mg/mL), while control cells received fresh medium. At the end of treatments, supernatants were discarded, and the cells were incubated with a solution of 0.5 mg/mL of MTT (Sigma-Aldrich) at 37 °C for 4 h. The formed formazan crystals were dissolved in 100 µL of a 0.1 N HCl/isopropanol solution. The absorbance was acquired at a wavelength of 570 nm (reference at 690 nm), using a microplate reader (Bio-Rad Laboratories, Milan, Italy). Results were reported as a percentage of cell viability, compared to untreated cells, which were set as 100%. The experiments were performed in eight replicates and repeated three times.

### 2.4. Assessment of Apoptosis

The involvement of apoptosis was investigated by annexin V-fluorescein isothiocyanate (FITC)/propidium iodide (PI) staining, as reported in [21]. Briefly, HCT-116 cells were seeded in 6-well plates (2.5 × 10^5^ cells/well) and, after 24 h, treated with the BJe (0.5–2.5 mg/mL). At the end of the treatments (24, 48, and 72 h), the harvested cells were resuspended in 200 µL of calcium-rich binding buffer, following kit guidelines (BD Biosciences, Milan, Italy). Then, 5 μL of Annexin V-FITC solution was added to each sample, and incubated for a further 15 min. Then, cells were resuspended in 10 µL of PI plus 190 µL of binding buffer. Samples were analyzed by a Novocyte 2000 flow cytometer (ACEA Biosciences Inc., Agilent, San Diego, CA, USA). Three independent experiments were performed, in which at least 10,000 events were analyzed.

### 2.5. Cell Cycle Analysis

The rate of cells populating each phase of the cell cycle was evaluated by PI staining, as shown in [22]. In brief, HCT-116 cells were seeded in 6-well plates at a concentration of 2.5 × 10^5^ cells/well. After 24 h, cells were treated for 24, 48, and 72 h with the BJe (0.5–2.5 mg/mL). Then, cells were harvested, washed with PBS, and fixed with 70% ice-cold ethanol overnight at 4 °C. Then, after a washing step with ice-cold PBS, cells were resuspended in 250 μL of PBS with 5 μL of RNase A (10 mg/mL; Sigma-Aldrich) and maintained for 1 h at 37 °C. Lastly, 10 µL of PI (1 mg/mL) was added to cells, and events were acquired by a Novocyte 2000 cytofluorimeter. Three independent experiments were performed, in which at least 10,000 events were analyzed.

### 2.6. Real-Time PCR

For the gene expression studies, HCT-116 cells were seeded in 100 mm Petri dishes (at a concentration of 1 × 10^6^ cells/dish) and treated with BJe (0.5–2.5 mg/mL) for 24 h at 37 °C. Afterwards, RNA extraction from untreated and treated cells was performed employing PureZol reagent (Euroclone, Milan, Italy), following the manufacturer’s protocol. The same quantity of total RNA (2 µg) for each sample was reverse transcribed into cDNA using the High-Capacity cDNA Archive Kit (Applied Biosystems, Life Technologies, Foster City, CA, USA), as described in [21]. Then, quantitative PCR reactions (qPCR) were performed by a 7500 qPCR System (Applied Biosystems) using SYBR^®^ Select Master Mix (Applied Biosystems), 0.2 µM of primers, and 25 ng of cDNA. Primer sequences employed for qPCR are listed in Table 1. Data were analyzed using the 2^−∆∆CT^ relative quantification method, compared to the endogenous control, namely β-actin (ACTB). The values are shown as n-fold change with respect to untreated cells.

### 2.7. Determination of Reactive Oxygen Species (ROS) and Mitochondrial Membrane Potential (ΔΨm)

Reactive oxygen species (ROS) and mitochondrial membrane potential (ΔΨm) were assessed fluorometrically. For both experiments, HCT-116 cells were seeded on 96-well plates (2.5 × 10^4^ cells/well) and treated with BJe (0.25–2.5 mg/mL) for 24 h.

The quantification of ROS was obtained by using 2′,7′-dichlorodihydrofluorescein diacetate (DCFH-DA; Sigma-Aldrich) while alterations of ΔΨm were estimated by quantifying rhodamine 123 (R123; Sigma-Aldrich) incorporation, as shown in [23]. The fluorescence was acquired by a microplate reader (POLARstar Omega, BMG Labtech, Ortenberg, Germany) at 485 nm excitation and 535 nm emission for DCFH-DA, while for R123 at 488 nm excitation and 525 nm emission. Results were expressed as a percentage of detected fluorescence, compared to untreated cells, set as 100%. All experiments were performed in eight replicates and repeated three times.

### 2.8. Cytofluorimetric Evaluation of DNA Damage

The oxidative damage to DNA was evaluated as levels of 8-oxo-2′-deoxyguanosine (8-oxo-dG) utilizing the FITC-labeled avidin probe (Sigma Aldrich). In brief, HCT-116 cells were permeabilized with ice-cold methanol and kept for 15 min at −20 °C. Then, samples were washed and incubated with avidin-FITC conjugate (0.2 μM) for 1 h. The fluorescence of 10,000 events was acquired with a Novocyte 2000 cytofluorimeter (495 nm excitation, 520 nm emission) [19].

The rate of DNA damage was also assessed as the level of phosphorylation of gamma-histone H2A.X (γ-H2A.X) employing the FITC-conjugated antibody (Merck-Millipore, Darmstadt, Germany). Briefly, HCT-116 cells were permeabilized with ethanol at −20 °C for 2 h and, after PBS washing, incubated with anti-phospho-γ-H2A.X (Ser139)-FITC conjugated for 1 h. The fluorescence of 10,000 events was recorded with a Novocyte 2000 cytofluorimeter at 495 nm excitation and 520 nm emission [24].

### 2.9. Animals

Male CD1 background mice (6 weeks old) were purchased from Charles River (Sant’Angelo Lodigiano, Italy) and housed in the conventional animal house of the Department of Pharmacy, University of Naples Federico II (Naples, Italy). All the animals were housed in polycarbonate cages supplemented with environmental enrichment under a 12-h light/12-h dark cycle, with a temperature of 23 ± 2 °C and 60% humidity. Animals were fed with standard food and water ad libitum. All efforts were made to minimize the number of animals used and their suffering. Specifically, mice were monitored daily for general health status, body weight, behavior, and appearance. Signs such as >15% body weight loss, severe lethargy, hunched posture, ruffled fur, or reduced food and water intake were considered humane endpoints, and animals meeting these criteria were humanely euthanized. Animals were randomly allocated to control and treatment groups by manual drawing of numbered cards to ensure unbiased group assignment. To minimize potential confounders, cages were rotated weekly within the animal room to avoid location bias, and treatments and sample collections were performed at the same time of day for all groups. Experimental procedures and protocols were in conformity with national (Direttiva 2010/63/UE) laws and policies and were approved by the Italian Ministry of Health.

### 2.10. Azoxymethane (AOM) Model of Colon Cancer

Azoxymethane (AOM) (40 mg·kg^−1^) (Cat. A5486, Sigma-Aldrich, Milan, Italy) was administered intraperitoneally (i.p.) in mice at a single dose of 10 mg·kg^−1^ once per week for 4 weeks. BJe (10–60 mg/kg) was administered by oral gavage three times per week for the whole duration of the experiment, starting 1 week before the first administration of AOM. All animals were humanely euthanized by asphyxiation with CO_2_ 12 weeks after the first injection of AOM. Aberrant crypt foci (ACF), polyps, and tumors were evaluated in mice colons as previously described [25]. Briefly, the colons were quickly removed after euthanasia, washed, opened longitudinally, laid flat, and fixed with 10% buffered formaldehyde. After staining with 0.2% methylene blue in saline, the colons were examined for the detection of ACF, polyps, and tumors using a light microscope at 20× magnification (Leica Microsystems, Milan, Italy). The investigator who allocated animals to groups was aware of the treatment assignments. However, experimenters responsible for data collection and outcome assessment were blinded to group identity.

### 2.11. Statistical Analysis

Results of three independent experiments performed in replicates are shown as mean ± standard error of the means (SEM). The statistical assessment for differences was carried out using one-way analysis of variance (ANOVA), employing Dunnett’s multiple comparison test (GraphPad Prism Software for Science, version 8.4.2, San Diego, CA, USA). The chi-square test was used to assess the significance of the difference in the number of mice with or without polyps or tumors. *p*-values less than or equal to 0.05 were considered significant.

## 3. Results

### 3.1. Quali-Quantitative Composition of BJe

**Figure 1:**
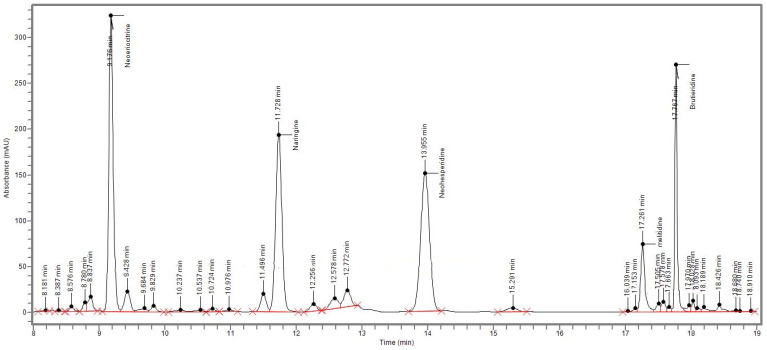
Representative chromatogram of the flavonoid profile of BJe. High-performance liquid chromatography coupled with a diode array detector (HPLC-DAD) separation of flavonoids present in BJe was recorded at 280 nm. The identification of peaks was carried out by comparing retention time and spectra of the flavonoids with pure reference molecules.

The quali-quantitative composition of BJe, assessed via HPLC (Figure 1), showed that the main flavonoids present in the extract employed in this study are naringin (116.2 ± 2.1 mg/g), neoeriocitrin (110.8 ± 1.6 mg/g), neohesperidin (94.8 ± 1.2 mg/g), brutieridin (46.5 ± 1.5 mg/g), and melitidin (24.2 ± 0.5 mg/g; Table 2).

### 3.2. BJe Inhibited the Proliferation of Different Colorectal Cancer Cells

**Figure 2:**
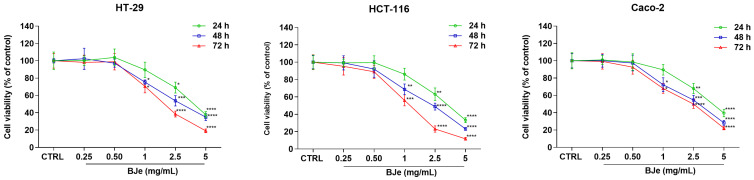
Effect of BJe (0.25 to 5 mg/mL) on the cell growth of HT-29, HCT-116, and Caco-2 lines for 24–72 h assessed by MTT test. Results are expressed as percentages ± standard error of the means (SEM) of the absorbance values detected in the control (CTRL) cells. Each concentration was tested eight-fold, and three independent experiments were carried out (*n* = 24). * *p* < 0.05, ** *p* < 0.01, *** *p* < 0.001 and **** *p* < 0.0001 vs. CTRL.

The effect of BJe on cell proliferation was assessed by the MTT test, as an index of mitochondrial respiration (Figure 2). As shown in Figure 2, BJe was able to hamper the viability of HT-29, HCT-116, and Caco-2 cells to different extents.

Indeed, the concentration of 2.5 mg/mL significantly hindered the proliferation of each cell line after 24 h of treatment. Whereas the 1 mg/mL displayed significant inhibition of viability after 48 h of treatment. The extrapolated IC_50_s at 72 h of treatment were 1.92 ± 0.47, 1.26 ± 0.28, and 2.19 ± 0.57 mg/mL for HT-29, HCT-116, and Caco-2 cells, respectively. These results suggested that the most sensitive cell line was the HCT-116 one. Therefore, the following experiments were performed specifically on this cell line.

### 3.3. BJe Induced Apoptosis in HCT-116 Cells

**Figure 3:**
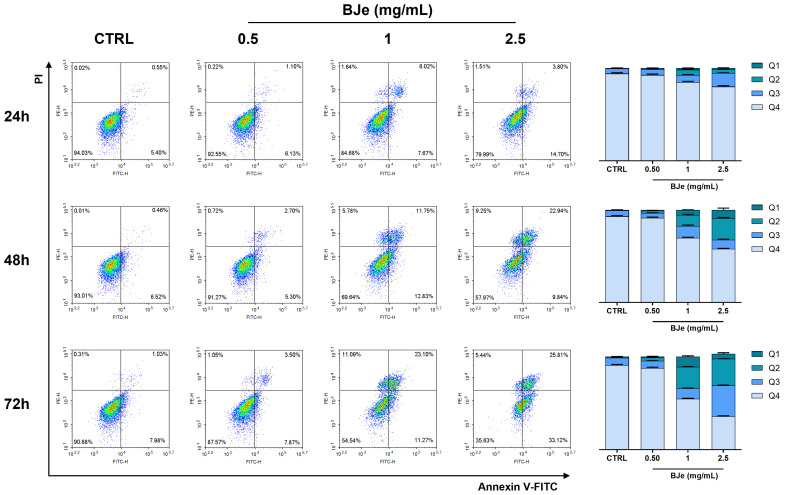
Analysis of apoptosis in HCT-116 cells treated with BJe. The detection of apoptosis was performed by annexin V-FITC/PI staining. Representative annexin V/PI dot plots of HCT-116 cells treated with BJe (0.5–2.5 mg/mL) for the indicated periods are shown. Q4 contains viable cells (lower-left quadrant), Q3 contains cells in early apoptosis (lower-right quadrant), Q2 contains cells in late apoptosis (upper-right quadrant), whereas Q1 contains necrotic ones (upper-left quadrant). Histograms describe the percentages of cells present in the corresponding quadrants ± SEM of three experiments performed separately in triplicate (*n* = 9).

To determine the type of cell death caused by BJe in HCT-116 cells, the annexin V-FITC/PI staining was employed to assess the potential involvement of apoptosis. The treatment with BJe at 1 and 2.5 mg/mL increased the percentage of cells undergoing both early and late apoptosis up to 14.6 ± 0.9% and 18.7 ± 0.2%, respectively, already at 24 h. These effects were markedly stronger at 48 h, in which BJe at 1 and 2.5 mg/mL pushed cells to undergo both early and late apoptosis up to 26.7 ± 2.1% and 34.4 ± 1.6%, respectively. Similarly, 1 and 2.5 mg/mL BJe augmented cells in both early and late apoptosis up to 34.9 ± 0.5% and 59.5 ± 0.6%, respectively. The concentration of 0.5 mg/mL of BJe was not able to induce any sign of apoptosis at any of the timings assessed (Figure 3).

### 3.4. BJe Blocked Cell Cycle in HCT-116 Cells

**Figure 4:**
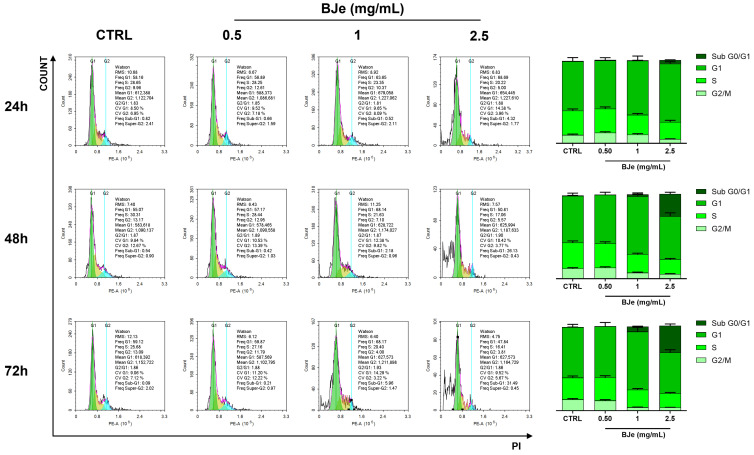
Effect of BJe on cell cycle progression of HCT-116 cells. Propidium iodide staining was employed to discriminate cells populating cells in each phase. The plots are representative of three different experimental sessions performed in triplicate (*n* = 9). Percentages ± SEM of cells present in each phase of the cell cycle are displayed in the histograms.

The effect of BJe on the cell-cycle progression of HCT-116 cells was evaluated by PI staining to discriminate cells in each phase (Figure 4). The treatment with BJe at concentrations of 1 and 2.5 mg/mL was able to augment cells in G1 phase up to 69.1 ± 5.5% and 70.9 ± 2.2%, respectively, compared to control cells (62.4 ± 4.2%), after 24 h. As for apoptosis, these effects were stronger at 48 h, where BJe 1 mg/mL increased cells in G1 phase up to 72.2 ± 4.1% compared to control cells (58.3 ± 3.2%). Interestingly, BJe 2.5 mg/mL augmented the number of cells in sub-G0/G1 phase (i.e., 28.2 ± 2.1% vs. 0.5 ± 0.01% of control cells), commonly referred to as hypodiploid cells exiting the cell cycle, and undergoing apoptosis. For BJe 1 mg/mL, we also observed a slight increase in cells in the sub-G0/G1 phase. This outcome was also reflected at 72 h of treatment, where both 1 and 2.5 mg/mL of BJe pushed cells out of the cell cycle, as demonstrated by the percentages of cells populating the sub-G0/G1 phase (i.e., 6.4 ± 0.4% and 33.4 ± 1.9%, respectively). Notably, BJe 1 mg/mL was able to maintain a high percentage of cells in G1 phase (i.e., 73.1 ± 4.9% compared to 52.3 ± 3.2% of control cells). The 0.5 mg/mL concentration demonstrated that it was not able to alter the cell cycle of HCT-116 cells at any of the tested timings (Figure 4).

### 3.5. BJe Modulated the Expression of Apoptotic- and Cell Cycle-Related Markers in HCT-116 Cells

**Figure 5:**
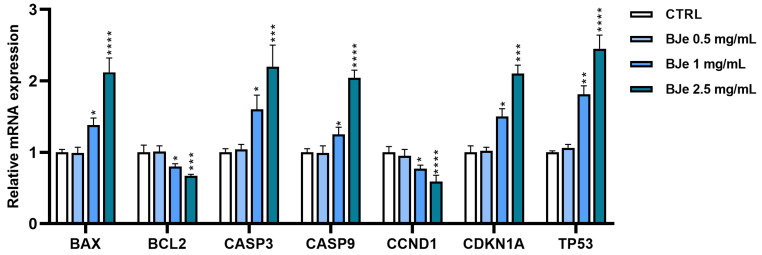
Modulation of apoptotic- and cell cycle-related gene levels in HCT-116 cells treated with BJe. Cells exposed to BJe (0.5–2.5 mg/mL) for 24 h were processed for mRNA expression studies. Relative quantities of mRNA, acquired by real-time PCR made in triplicate, were calculated by the 2^−∆∆Ct^ method, with β-actin (ACTB) as housekeeping gene. Results are expressed as fold change with respect to untreated cells and expressed as mean ± SEM of three different experiments performed in triplicate (*n* = 9). * *p* < 0.05, ** *p* < 0.01, *** *p* < 0.001 and **** *p* < 0.0001 vs. CTRL.

Based on the observed effects of BJe in HCT-116 cells, we investigated the potential factors involved by means of gene expression studies. The treatment for 24 h with BJe 1 and 2.5 mg/mL brought a modulation of both apoptotic and cell cycle-related markers in a concentration-dependent fashion. Indeed, both BJe 1 and 2.5 mg/mL prompted an increase in the expression of pro-apoptotic markers B-cell lymphoma-2 (BCL2)-associated X apoptosis regulator (BAX) up to 38 ± 2.75% and 112 ± 10.6%, respectively, and a decrease in the expression of the anti-apoptotic BCL2 of 20 ± 1% and 33 ± 0.99%, respectively, compared to control cells. This was coupled with an increased expression of the caspase-3 (CASP3) of 60 ± 7.5% and 120 ± 16.4%, as well as caspase-9 (CASP9) of 25 ± 2% and 104 ± 5.61% induced by the 1 and 2.5 mg/mL of BJe, respectively. Moreover, the treatment with BJe unleashed a dramatic increase in p53 (TP53). Indeed, BJe 1 and 2.5 mg/mL pushed the expression of TP53 up to 81 ± 5.37% and 145 ± 11.24%, respectively, compared to control cells. This modulation was also reflected in cell cycle-related factors, such as p21 (CDKN1A) and cyclin D1 (CCND1). Treatment with BJe at both 1 and 2.5 mg/mL increased the expression of CDKN1A up to 50 ± 3.67% and 110 ± 6.29%, respectively, which was linked to a decrease in the expression of CCND1 of 23 ± 1.49% and 41 ± 6.25%, respectively, compared to control cells (Figure 5).

### 3.6. BJe Enhanced Reactive Oxygen Species (ROS) and Impaired Mitochondrial Membrane Potential (∆Ψm)

**Figure 6:**
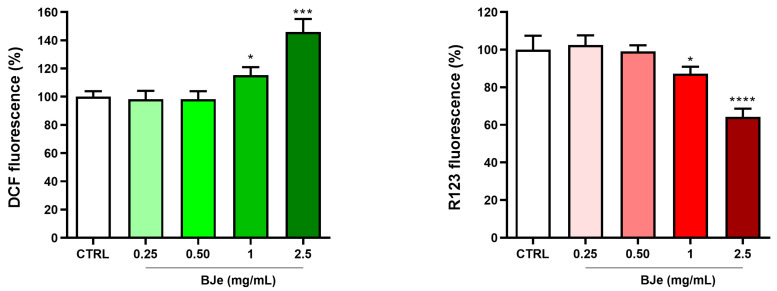
Level of ROS and ΔΨm in HCT-116 cells after treatment with BJe (0.25–2.5 mg/mL). ROS levels were evaluated through the fluorescent probe DCFH-DA (in green), while ΔΨm variations were assessed through R123 staining (in red) after 24 h of treatment. Results of both assays are expressed as percentages ± SEM of the fluorescence values detected in the control cells of three different experiments performed in eight replicates (*n* = 24). * *p* < 0.05, *** *p* < 0.001 and **** *p* < 0.0001 vs. CTRL.

The production of ROS in HCT-116 cells treated with BJe (0.5–2.5 mg/mL) for 24 h was evaluated by staining with DCFH-DA. As shown in Figure 6, BJe at both 1 and 2.5 mg/mL was able to significantly raise ROS production in HCT-116 cells up to 115.2 ± 5.8% and 145.9 ± 9.2%, respectively. In parallel, employing the R123 staining, it was observed that BJe hampered ∆Ψm. Indeed, BJe 1 and 2.5 mg/mL significantly decreased the ∆Ψm of treated HCT-116 to 87.2 ± 3.8% and 64.25 ± 4.4%, respectively (Figure 6).

### 3.7. BJe Induced DNA Damage in HCT-116 Cells

**Figure 7:**
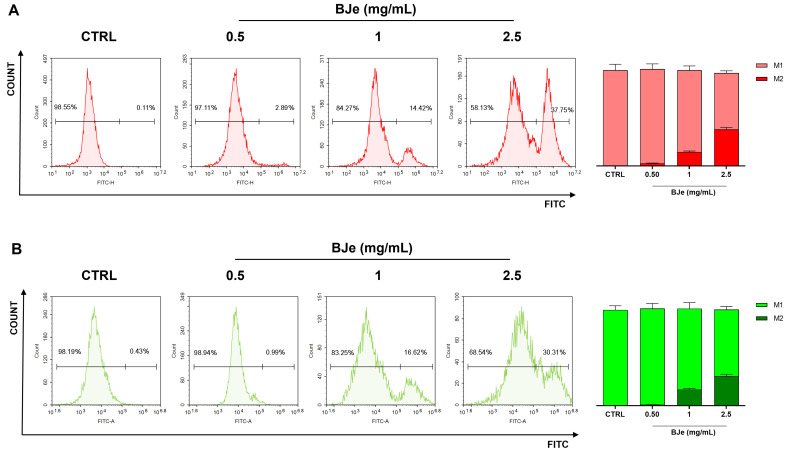
Cytofluorimetric evaluation of DNA damage in HCT-116 treated with BJe (0.5–2.5 mg/mL). Levels of 8-oxo-dG were measured as fluorescence of the FITC-conjugated avidin ((**A**); in red). Levels of phosphorylated gamma-H2A.X were assessed as fluorescence of the FITC-conjugated antibody ((**B**); in green). Plots are representative of three separate experiments. The histograms represent the percentage ± SEM of normal cells (non-fluorescent, M1) and damaged ones (fluorescent, M2) of three independent experiments, performed in triplicate (*n* = 9).

The effects of BJe treatment in HCT-116 cells in terms of DNA damage were assessed by quantifying the oxidized nucleobase derivative, namely 8-oxo-dG, and phosphorylation of γ-H2AX. As shown in Figure 7A, BJe 1 mg/mL induced an increase in levels of fluorescent cells in M2, meaning higher quantities of 8-oxo-dG, up to 15.4 ± 1%, whereas BJe 2.5 mg/mL up to 39.8 ± 2.1%. This effect was coupled with an increase in DNA damage associated with phosphorylation of γ-H2A.X. Indeed, Bje at 1 mg/mL prompted an increase in levels of phospho-H2A.X up to 17.6 ± 1.05%, while Bje at 2.5 mg/mL up to 32.4 ± 2.1% (Figure 7B).

### 3.8. Effect of BJe in AOM Model of Colon Cancer

**Figure 8:**
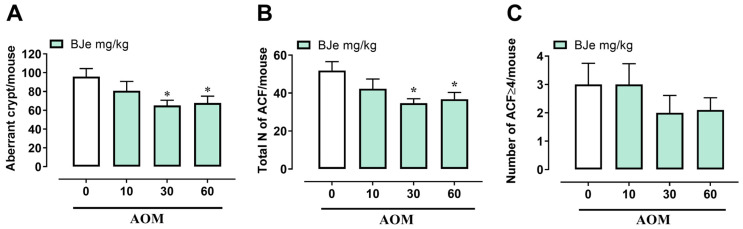
Effect of BJe treatment on the murine AOM model of colon cancer. Number of aberrant crypt (**A**), total number of aberrant crypt foci (ACF) (**B**), and number of ACF containing more than four crypts (**C**) derived from the administration of AOM (40 mg/kg, i.p.). BJe (10–60 mg/kg) was given three times a week for the whole duration of the experiment, starting 1 week before the first administration of AOM. Measurements were performed 3 months after the first injection of AOM. Results represent the mean ± SEM of 10 mice. * *p* < 0.05 vs. AOM alone.

**Figure 9:**
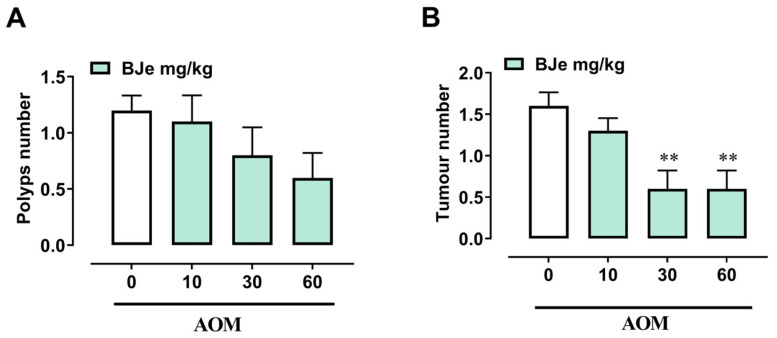
Effect of BJe on the formation of polyps (**A**) and tumors (**B**) induced in the mouse colon by the carcinogenic agent AOM (40 mg/kg, i.p.). BJe (10–60 mg/kg) was given three times a week for the whole duration of the experiment, starting 1 week before the first administration of AOM. Measurements were performed 3 months after the first injection of AOM. Results represent the mean ± SEM of 10 mice. ** *p* < 0.01 vs. AOM alone.

In order to investigate the antitumoral role of BJe in vivo, we used the AOM model of colon cancer, a well-established murine model for studying tumor initiation and progression. AOM injection for four consecutive weeks resulted in a slight decrease in body weight compared to control mice. Pretreatment with BJe (10–60 mg/kg) did not significantly alter body weight compared with AOM alone. Similarly, BJe at the highest dose (60 mg/kg) given alone (without AOM) did not induce changes in body weight compared to the control. Consistent with the expected pathological spectrum of this model, 100% of AOM-treated mice developed polyps and tumors (Table 3). Pretreatment with BJe, at the doses of 30 and 60 mg/kg, significantly decreased the percentage of mice developing polyps and tumors (Table 3). BJe pretreatment also reduced (i) the number of aberrant crypts, (ii) the total number of aberrant crypt foci (ACF), and (iii) the total number of tumors per mouse (Figure 8A,B and Figure 9B). There was only a trend towards reducing ACF containing more than four crypts and the number of polyps (Figure 8C and Figure 9A). These findings demonstrate that BJe administration exerted a chemopreventive effect by effectively reducing colon cancer development.

## 4. Discussion

For early-stage CRC patients, capecitabine and oxaliplatin or irinotecan 5-fluorouracil are the medications most frequently used to treat this type of cancer. As the pathology progresses, the main strategy for patients with advanced CRC is surgical resection, which is followed by chemotherapy or radiation therapy to eradicate any remaining cancer cells [26]. Finding novel, efficient medications with fewer side effects is crucial for treating CRC because of drug resistance and the severe side effects of these traditional chemotherapies, which include cardiotoxicity, neurotoxicity, hair loss, bone marrow suppression, and gastrointestinal disturbances [27]. For these reasons, the preventive properties against cancer of naturally occurring compounds derived from fruits and vegetables that are frequently included in diets have always ignited a rising interest [28,29]. Among these, CF and their derivatives have been suggested as co-adjuvants in cancer therapy since the scientific community has long endorsed their potential as antitumor agents. This is because CF contains plenty of flavonoids, which have been shown to be multitarget agents able to interfere with several pathogenic pathways [30], also in the field of CRC [31,32]. In this frame, bergamot derivatives have been demonstrated to possess anti-cancer properties, among others. Indeed, we observed that BJe and its flavanones inhibited the proliferation of monocytic leukemia cells via the inhibition of SIRT2, a histone deacetylase claimed to be a prognostic marker in acute myeloid leukemia [21,22]. In the field of CRC, we demonstrated that BJe was able to hamper the proliferation of HT-29 cells [19]. Moreover, BJe prevented spontaneous tumorigenesis in a genetic model of CRC, namely Pirc rats (F344/NTac-Apcam1137), via its anti-inflammatory and pro-apoptotic effects [18].

Based on these premises, in this study, we aimed to investigate the mechanisms underlying the anticancer effects of BJe in CRC experimental models. Firstly, we confirmed the quali-quantitative composition of the extract. Indeed, we found that BJe was almost exclusively composed of naringin, neoeriocitrin, and neohesperidin, flavonoids typical of the *Citrus* genus, along with brutieridin and melitidin, which are characteristic of the bergamot fruit [33,34]. Nevertheless, we cannot neglect the potential contribution of the flavonoid present in trace amounts in BJe. Indeed, it is acknowledged that the potential of natural matrices as a source of compounds with remarkable pharmacological effects lays in the combination of compounds present in phytocomplexes, which may act synergistically to convey a stronger response than the one achievable employing a single compound alone [35].

For the in vitro studies, we chose concentrations spanning from 0.25 to 5 mg/mL based on previous reports [19,22]. As expected, BJe was able to hamper the proliferation of HT-29 cells, as well as that of Caco-2 cells. It is interesting to note that BJe also inhibited the proliferation of HCT-116 cells to a greater extent than observed in the other two cell lines used, as confirmed by the lower IC_50_ values we obtained. Therefore, we continued the study by employing this cell line. These effects are in line with other reports that have claimed that naringin, the main flavonoid present in BJe, reduced the proliferation of several CRC cell lines, among which HCT-116 [36,37]. Moreover, CF waste rich in flavonoids, like neohesperidin and neoeriocitrin, has also been shown to hamper the proliferation of HCT-116 cells, thus supporting our findings [38].

In order to investigate the type of death induced by BJe in HCT-116, we assessed whether apoptosis was involved. By cytofluorimetric analyses, we found that BJe induced both early and late apoptosis in this cell line in a concentration- and time-dependent fashion. Moreover, gene expression studies have supported our results since BJe was able to increase the expression of pro-apoptotic markers (i.e., BAX, TP53, CASP3, and CASP9) and decrease that of anti-apoptotic ones (i.e., BCL2). In the literature, naringin and its aglycone have been reported to induce apoptotic cell death in HCT-116 cells, as well as in other CRC cell models, along with alter the factors regulating this process [36,37,39].

Since another mechanism of cell death might be the influence on cell-cycle progression, we then focused on this aspect to fully unravel the mechanisms through which BJe acted. Indeed, the treatment of HCT-116 cells with BJe showed a robust increase in cells populating G1 phase, meaning a blockage at this stage, with a consequent remodulation of the percentages in other phases. Interestingly, at increasing exposure and concentration, cells exited the cell cycle, thus populating the sub-G0/G1 phase, typically that of hypodiploid and apoptotic cells. Moreover, this was confirmed by the modulation of factors regulating this stage of the cell cycle, namely p21 and cyclin D1. The former is known to typically bind to the CDK complex, inhibiting its kinase activity and stopping cell-cycle progression during the G1 phase [40]. Of note, other polyphenols have shown the capacity of blocking the cell cycle of HCT-116 cells at this stage, with a regulation of the abovementioned markers. Indeed, epigallocatechin-3-gallate, naringenin, and neohesperidin down-regulated the levels of both cyclin D1 and increased those of p21 in different human CRC cell lines [41,42,43].

At this point, we wondered which mechanism could lie beneath BJe’s pro-apoptotic and cell cycle-blocking effects in HCT-116 cells. This cell line is characterized by a deficiency in MutL protein homolog 1 (MLH1) protein, which is a DNA mismatch repair protein whose absence is associated with the microsatellite instability witnessed in some forms of CRC [44]. MLH1 is linked to dysregulated mitochondrial metabolism, lower basal oxygen consumption rate, and decreased residual respiratory capacity. In addition, MLH1-deficient cells have significantly reduced respiratory chain Complex I activity. As a result of this altered mitochondrial metabolism, MLH1-deficient cells have a lower antioxidant capacity and are more sensitive to ROS-inducing compounds [45]. For these reasons, we assessed the redox status in HCT-116 cells treated with BJe. Notably, our extract was able to increase the levels of intracellular ROS in a concentration-dependent fashion, an event that correlated with the loss of the mitochondrial membrane potential. In this line, naringin, combined with epicatechin, has been shown to prompt oxidative stress in CRC cells, like HCT-116 ones [46].

It is acknowledged that a high level of ROS has the ability to disrupt DNA and alter the DNA damage response. Moreover, there is strong evidence that ROS support the genotoxic stress brought on by both ionizing therapy and chemotherapeutic drugs. In detail, it has been revealed that the mechanisms by which ROS might affect the cellular reaction to genotoxic therapy-induced DNA damage involving double-strand breaks (DSBs) [47]. Although the exact link between ROS and phosphorylation of γ-H2A.X is not fully understood, it is known that acute oxidative stress is able to increase γ-H2AX activation, which in turn is fundamental for the recruitment of multiple components of the DNA damage response to the site of DNA DSBs to initiate the repair [48]. In our study, this increased oxidative stress induced by BJe has led to an elevated presence of oxidized nucleobases at the level of DNA, as proved by the progressive increase of 8-oxo-dG levels as BJe concentrations augmented. This was coupled with the phosphorylation status of γ-H2A.X, which demonstrated the activation of repair mechanisms towards DNA damage.

The genomic instability we observed in cells is an important molecular event occurring also in rodent models. In particular, AOM-injected mice are characterized by genetic changes that contribute to the development of colon carcinogenesis. For example, TGF- β, β-catenin, and K-ras mutations have been identified to be caused by single-nucleotide changes, which appear to be common and frequent modifications in this model. When DNA replicates, this carcinogen produces the methylated DNA adduct O-6-methylguanine, which can mispair with thymidine and cause unfixed transitions [49,50].

Using this mouse model of sporadic CRC, we demonstrated that BJe significantly and dose-dependently reduced the percentage of mice developing polyps and tumors. In addition, BJe significantly decreased the number of aberrant crypts, the total number of aberrant crypt foci, and the total number of tumors per mouse. Although the reduction in precancerous lesions, such as ACF, with four or more crypts and polyps did not reach statistical significance, a clear downward trend was observed. Overall, these findings indicate that BJe treatment effectively inhibited the initiation and progression of tumors induced by the carcinogenic agent AOM, thereby confirming in vivo the anticancer properties previously demonstrated in HCT-116 cells. Other studies have reported the properties of other CF derivatives or flavonoids in counteracting AOM-induced carcinogenesis. Indeed, both naringin and neohesperidin protected against CRC in mice, as well as the correlated colitis, via suppression of the NF-κB/p65 pathways [51,52].

## 5. Conclusions

In this study, we showed that BJe possessed anti-proliferative effects in several CRC cell lines. Moreover, we observed that the induction of apoptosis and blockage of the cell cycle in HCT-116 cells may be ascribed to the increase in ROS production, which led to an augmentation of oxidative stress at the level of the nucleus and concomitant DNA damage. Furthermore, BJe lowered the presence of tumors in AOM-injected mice, as well as that of aberrant crypt foci. Despite the key limitations of both in vitro studies and animal models, these results provide preliminary evidence that could be relevant to human biology. In conclusion, our results strengthen the anti-cancer properties of BJe, supporting its potential role in the management of CRC, also when characterized by MSI.

## Figures and Tables

**Table 1:** Oligonucleotide primer sequences used for real-time PCR.

Gene Symbol	Gene Name	NCBI Reference Sequence	Primer Sequence
ACTB	β-actin	NM_001101.5	Forward: 5′-TTGTTACAGGAAGTCCCTTGCC-3′ Reverse: 5′-ATGCTATCACCTCCCCTGTGTG-3′
BAX	BCL2-associated X, apoptosis regulator	NM_138764.5	Forward: 5′-GGACGAACTGGACAGTAACATGG-3′ Reverse: 5′-GCAAAGTAGAAAAGGGCGACAAC-3′
BCL2	B-cell lymphoma-2	NM_000657.3	Forward: 5′-ATCGCCCTGTGGATGACTGAG-3′ Reverse: 5′-CAGCCAGGGAAATCAAACAGAGG-3′
CASP3	Caspase-3	NM_004346.4	Forward: 5′-AGCACCTGGTTATTATTCTTGG-3′ Reverse: 5′-GCTTGTCGGCATACTGTT-3′
CASP9	Caspase-9	NM_001229.5	Forward: 5′-GCTCAGACCAGAGATTCG-3′ Reverse: 5′-ATCCTCCAGAACCAATGTC-3′
CCND1	Cyclin D1	NM_053056.3	Forward: 5′-ACCTCTTCACCTTATTCAT-3′ Reverse: 5′-TTTGTCACTGGATGGTTT-3′
CDKN1A	p21	NM_000389.5	Forward: 5′-TTCTCCACCTAGACTGTAA-3′ Reverse: 5′-GCACCTGCTGTATATTCA-3′
TP53	p53	NM_000546.6	Forward: 5′-GTGTGGAGTATTTGGATGAC-3′ Reverse: 5′-ATGTAGTTGTAGTGGATGGT-3′

**Table 2:** Quali-quantitative composition of BJe. Values are expressed as mg/g ± SEM of dry weight of the extract.

Compound	mg/g (Dry Weight)
Brutieridin	46.5 ± 1.5
Melitidin	24.2 ± 0.5
Naringin	116.2 ± 2.1
Neoeriocitrin	110.8 ± 1.6
Neohesperidin	94.8 ± 1.2

**Table 3:** Effect of BJe (10–60 mg/kg) on the percentage of mice bearing polyps or tumors induced by the carcinogen agent AOM. * *p* < 0.05 and ** *p* < 0.01 vs. AOM alone.

	Number	Mice Bearing Polyps (%)	Mice Bearing Tumors (%)
Ctrl	10	0	0
BJe 60 mg/kg	10	0	0
AOM	10	100	100
AOM + BJe 10 mg/kg	10	80	100
AOM + BJe 30 mg/kg	10	60 *	50 **
AOM + BJe 60 mg/kg	10	50 **	50 **

## Data Availability

The original contributions presented in this study are included in the article. Further inquiries can be directed toward the corresponding author.

## Abbreviations

The following abbreviations are used in this manuscript:

ACF	Aberrant crypt foci
AOM	Azoxymethane
BJe	Bergamot juice extract
CF	Citrus fruits
CRC	Colorectal carcinoma
DAD	Diode array detector
FITC	Fluorescein isothiocyanate
HPLC	High-performance liquid chromatography
PI	Propidium iodide
ROS	Reactive oxygen species
